# Japanese Cross-ministerial Strategic Innovation Promotion Program “Innovative AI Hospital System”; How Will the 4th Industrial Revolution Affect Our Health and Medical Care System?

**DOI:** 10.31662/jmaj.2021-0133

**Published:** 2021-12-15

**Authors:** Yusuke Nakamura

**Affiliations:** 1 The Cross-ministerial Strategic Innovation Promotion Program “Innovative AI Hospital System”

**Keywords:** Artificial intelligence (AI), database, human errors, imaging, digital pathology

## Abstract

I introduce here what we have been and are doing in the Cross-ministerial Strategic Innovation Promotion Program (SIP) “Innovative AI Hospital System” project supported by the Japanese government. This SIP program began in April 2018 as 1 of 12 SIP projects that aim to establish “Society 5.0” in various scientific areas. This AI hospital program is the only one related to the health and medical care field, in which we are attempting to construct a big medical database, to implement many artificial intelligence (AI) tools in hospital systems, and to make the medical care system more effective along with reduction of workload of medical workers. To maintain or improve the quality of the health and medical care system, it is indispensable to share various updated and useful information among healthcare workers as well as the general population. We have been challenging to establish the digitalized medical care system in various aspects. I describe here where we are and the future perspective of the AI-based digital medicine.

## Introduction

The Cross-ministerial Strategic Innovation Promotion Program (SIP) is a national program that is led by the Council for Science, Technology, and Innovation of the Japanese Government. SIP is aiming to apply interdisciplinary management and cause scientific and technological innovation in Japan. SIP phase II began in 2018 (a total of approximately 200 million USD/year × 5 years), and it consists of 12 programs, which are trying to address critical social issues that Japan is facing, and is expected to resurge the Japanese economy. Technologies using artificial intelligence (AI) are considered to cause the fourth industrial revolution and influence various social and industrial areas. One of the SIP projects is aiming to revolutionize our medical and healthcare system by accelerating hospital digitalization and the implementation of AI tools (approximately 22-27 M USD/year for the first 4 years; the budget in the fifth year is not fixed).

**Figure 1.**
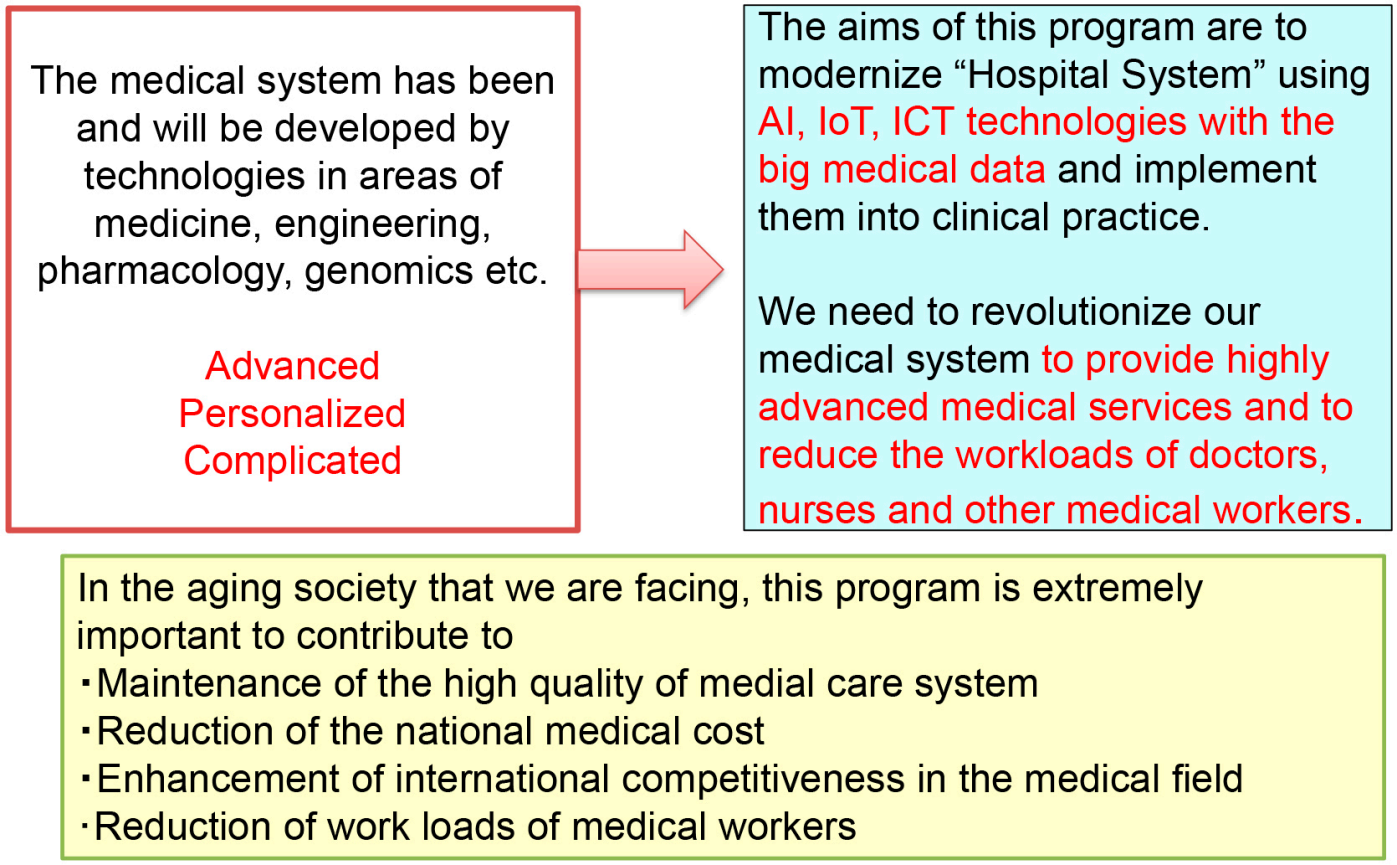
This summarizes the current issues to be solved in our medical care system and the aims of the SIP “AI Hospital” project.

Our medical care system is becoming extremely sophisticated, advanced, complicated, and personalized according to advances in many scientific areas, including medicine, engineering, pharmacology, and genomics. How to share highly advanced medical information among medical workers and transfer it to patients or the general population in an easy-to-understand manner is quite important for the maintenance or improvement of the quality of medical care in the future (Figure 1) ^(1)^. In addition, because our country is facing aging issues (the age of 29.1% of Japanese population is 65 years or higher), we need to avoid the steep elevation of the national medical cost, to keep our international competitiveness in medically related industries, and to reduce the workload of medical workers.

**Figure 2.**
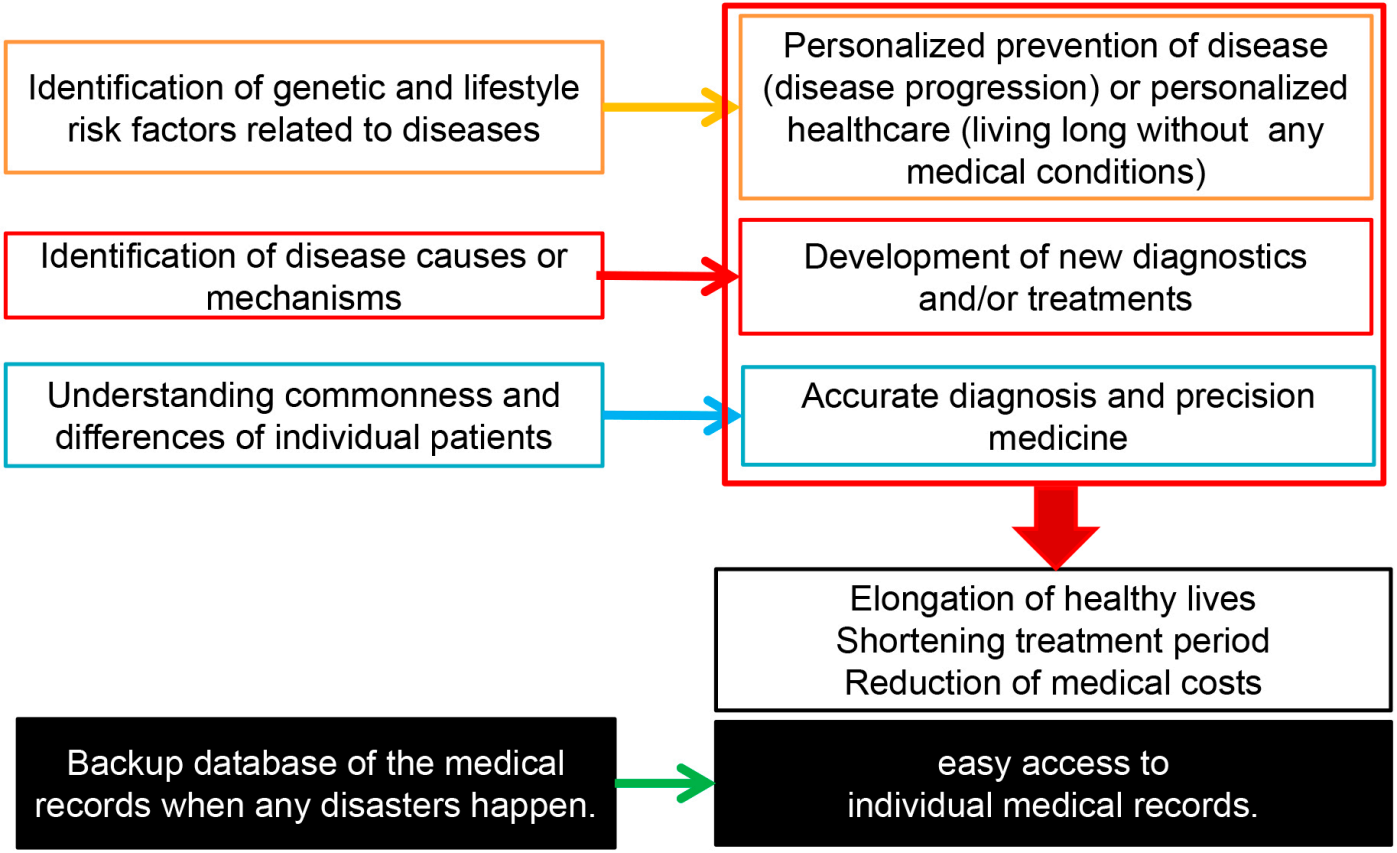
The reasons why we need to construct a big health and medical database.

In addition, construction of a big medical database, including personal health and medical records, is a critical and essential issue worldwide. The UK has a long-standing system for collecting prescription information, and Genomics England has been collecting medical records of a large number of individuals, including their genomic information ^(2)^. Why do we need a big medical database? ^(3)^ A large data set will help us understand the interactions between the environmental and genetic factors involved in various diseases. Such information can be applied for the prevention of illnesses as well as prevention of disease progression. However, we need to consider huge interindividual differences and establish a personalized approach. For example, if all adult people have cancer screening once a year, the capacity of the present medical care system is far below to cover everyone. Hence, it will be essential to personalize the contents of health examinations based on various information, including individual genetic makeup. Furthermore, we need to recognize interindividual diversity even if patients are diagnosed to have the same disease. Hence, we need to understand interindividual differences to treat patients more appropriately (Figure 2).

By creating a big database of health and medical information and applying deep learning with AI, I believe that it would be possible to establish precision medicine, to extend healthy life expectancy, to shorten treatment periods, to reduce medical expenses, and to keep a national labor force, all of which will solve major social issues, which Japan and other countries are and will be facing. If we can find answers to address these issues, we will also be able to help other nations, particularly those facing a rapid increase in elderly populations.

## AI Tools Needed in Medical Fields

I would like to introduce some examples of the AI tools or algorisms required in the medical field. Accurate diagnosis of radiological/ultrasound imaging and pathological pictures has become one of the especially critical issues in Japan. It has become evident that with deep learning using a vast number of images along with accurate clinical diagnostic information, AI can learn by itself, can find unique patterns, and can be applied as a diagnostic aid with a certain degree of accuracy. In Japan, the number of CT and MRI equipment per one million people is 5-6 times higher than that in other OECD countries, and that of radiologists per one million people is among the lowest in OECD countries ^(4)^. Considering the number of images produced in medical institutions in Japan, the capability seems to be beyond that of radiologists. Similarly, in the field of pathology, because the number of clinical pathologists has been decreasing, the number of doctors making the diagnosis seems to be much lower than that of slide glasses. Therefore, if AI can help or support the diagnosis in clinical pathology or in imaging at some extent, the number of cases requiring diagnosis with specialists will be reduced and AI can assure high-quality and accurate diagnosis throughout the world. In fact, an AI algorism, which can be applied for breast cancer screening with mammography, has been evaluated internationally ^(5)^. If AIs in many areas of imaging or clinical pathology are developed, the quality of medical care should be maintained anywhere in Japan and other countries.

**Figure 3.**
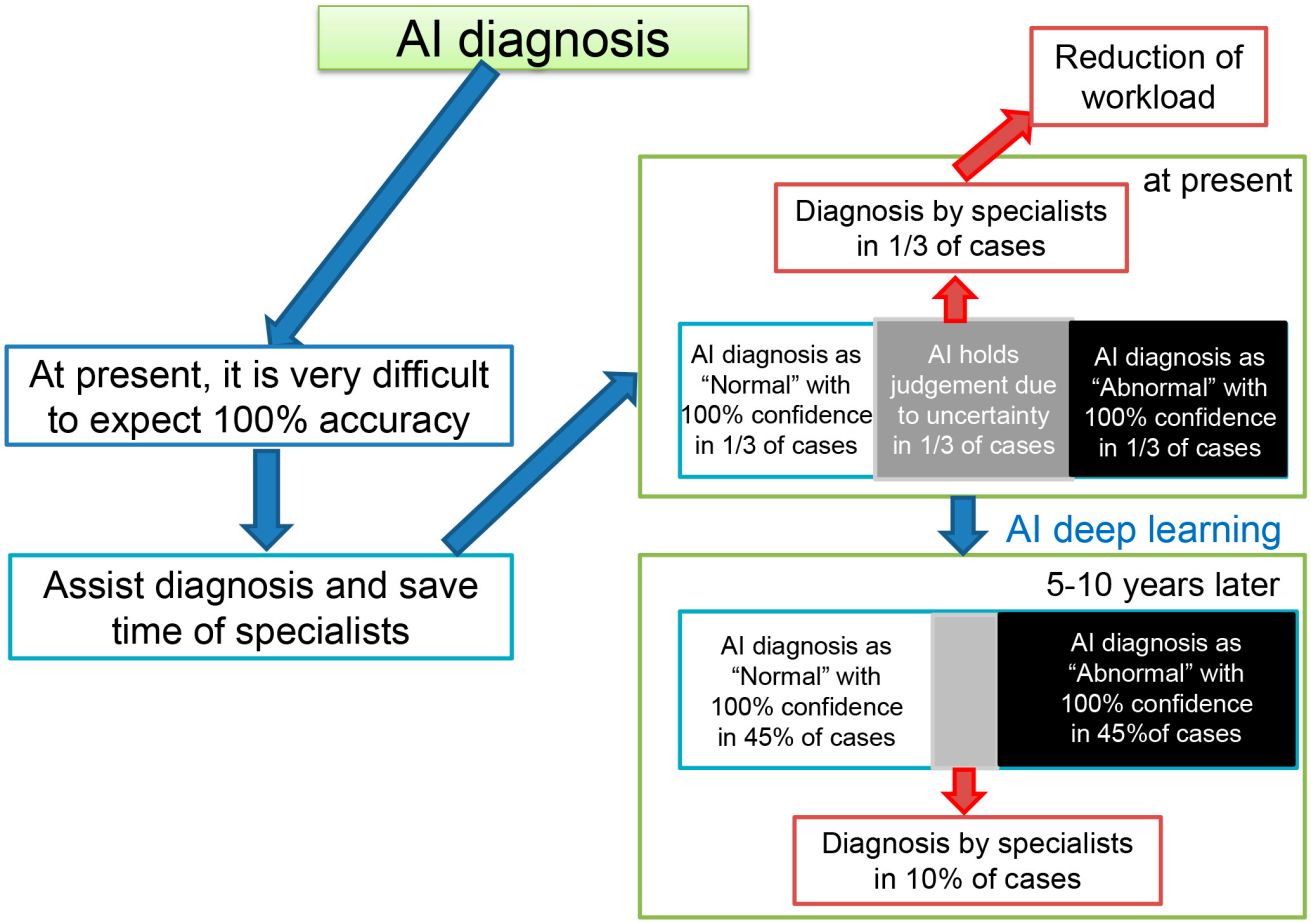
AI assistance of diagnosis in imaging pictures and digital pathology. AI may judge certainly normal and abnormal cases (one-third each), and professionals need to read the remaining one-third of the pictures for a while. However, through deep learning using thousands of images and digital pathological pictures with accurate annotations in the next 5-10 years, the accuracy of AI diagnosis will reach specialist levels.

However, the diagnosis through these AI algorisms has not yet reached the accuracy as many doctors or patients expect. Although it is one of the essential issues to discuss who should have responsibility to clinical diagnosis, doctors, including radiologists and clinical pathologists, at present need to have the final responsibility in diagnosis because we cannot fully rely on the accuracy of the diagnosis made by AIs. Hence, for a while, we should ask AIs to help in sorting out imaging films or histopathological slides in three categories (normal, questionable, and abnormal) as shown in Figure 3. If AI can provide some assistance, we can reduce the number of films or slides that can be judged by the eyes of specialists in radiology or clinical pathology. A questionable area (in a gray region), which AI cannot make the accurate diagnosis, can be reduced by further deep learning with high-quality annotations (true values) in the next 5-10 years, leading to the refinement of the AI algorisms and further reduction of workloads of radiologists or clinical pathologists. However, it may take a lengthy period (many years) that AI algorisms can achieve the extremely high accuracy (close to 100%) to make doctors or patients accept AI’s replacement of human diagnosis.

**Figure 4.**
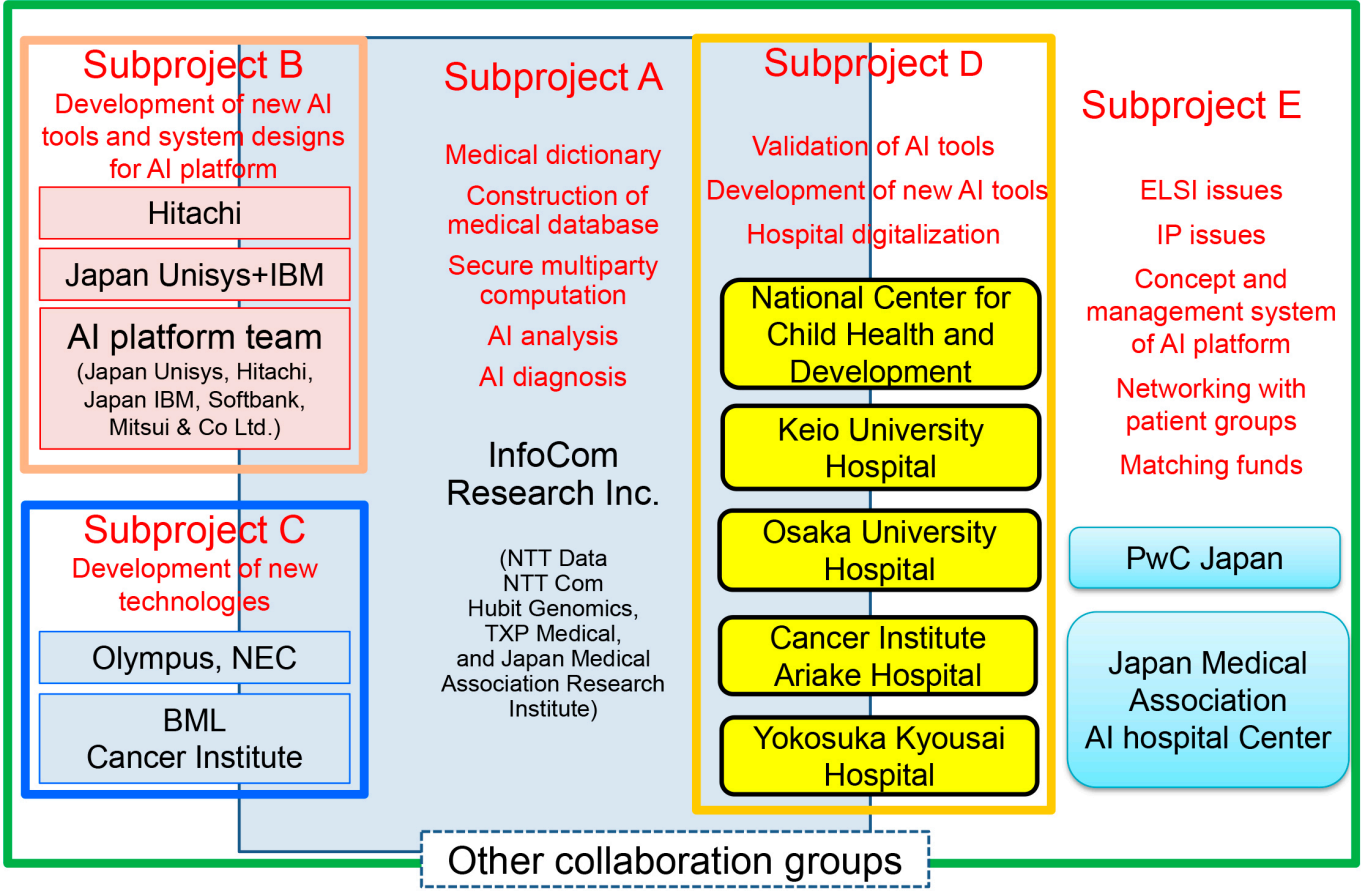
Various AI tools needed in clinical practices.

As shown in Figure 4, various AI tools or algorisms are expected to be implemented in daily clinical practices. For example, it is becoming possible for healthcare professionals, such as family doctors, to detect atrial fibrillation simply by collecting data using wearable devices and monitoring the pulse rhythm. The most advanced wearable device can measure electrocardiogram to find patients having atrial fibrillation or other types of arrythmia ^(6)^. It is well known that atrial fibrillation often leads to blood clotting and results in stroke. In Japan, the baby boomer generation who were born after World War II reached 70 years old or older. Because 1 in 20 to 30 people who are >70 years old is estimated to have atrial fibrillation, introduction of such wearable devices and digital reporting systems in the medical care system can gather risk for stroke and can provide earlier treatment opportunities to patients before cerebral infarction occurs. This kind of management system will reduce the number of patients who may have disability and require long-term care. In addition, AI, which can judge distinct types of arrythmia in the electrocardiogram using a deep neuronal network with the accuracy at the cardiologist level, was already reported ^(7)^. Under the corona pandemic situation, AIs that can detect apnea caused by opioid overdose using smartphones ^(8)^ should also be applied for monitoring the respiratory condition (hyperpnea) of corona-virus-infected individuals.

Furthermore, prescription errors, oversight of patients’ imaging data, and various other human errors are inevitable as we are human beings. However, if AI can recognize that a prescribed drug(s) is unmatched with the disease and can immediately let a doctor know it, the errors can be easily avoided. A report from the Network for Excellence in Health Innovation in the USA indicated that 7 million people were affected by prescription errors, resulting in ~7,000 deaths per year and unnecessary medical spending of ~40 billion USD ^(3)^. Hence, a digital system with AI is expected to avoid the consequent risk of prescription errors in patients, saves doctors from unexpected stresses, and reduces unnecessary medical costs. If it sends the information of his/her oversight to the attending physician immediately, AI should certainly avoid the delay of diagnosis and may save patients’ lives. Hospitals can easily avoid mis-serving meals in hospitals; providing meals containing allergens to allergic patients are sometimes deleterious and lethal.

## Automated AI Recoding in Medical Records and Nursing Records, and Assistance of Informed Consent Processes with AI

One of the key issues that are becoming more serious in the medical field is that doctors do not have enough time to talk to patients with eye contacts because they need to look at the keyboard and screen of the computer for entering medical records during the examination. According to a survey of medical institutions in this project, during a 10-min examination, doctors spent only 1-2 min to look at and talk to their patient. Considering the fundamental issue in medical care, insufficient time for looking at patients with eye contact reduces patients’ satisfaction. To solve this issue, we need to make AIs that can turn spoken languages into text and make summaries of medical records. Of course, such AIs are necessary in the nursing records and long-term care sites.

In addition, it is well recognized that a lot of time is spent in informed consent processes. To reduce the time burden on doctors, nurses, or research coordinators, AI avatars on screen could replace the stereotype and basic explanations, although it takes another 10-20 years for AIs to do bidirectional communication between patients and AI avatars. If AI does, patients and their family members can ask to explain repeatedly without any hesitation. After getting explanations from AIs, patients and their family members can discuss with their doctors/nurses in a face-to-face meeting and can make their decisions on the kind of treatment or a drug(s) they want to have. This kind of voice (spoken language)-to-text translation AIs will lead to work-style reform that is essential in the medical care system, particularly under the pandemic situation. In the United States, doctors have been quitting their jobs because of the burnout syndrome, partly due to spending a huge amount of time for explaining the medical procedures and/or responding to complaints, resulting in suicide in some cases. On the other hand, patients and their families feel that they are not sufficiently explained or are not well taken care of properly and feel frustrated. Hence, one of our goals in this project will be to replace the work, which can be performed without a doctor’s license or a nurse’s license, with AI as much as possible to be able to provide them more time for personalized medical care by directly facing their patients.

## AI Project Formation

**Figure 5.**
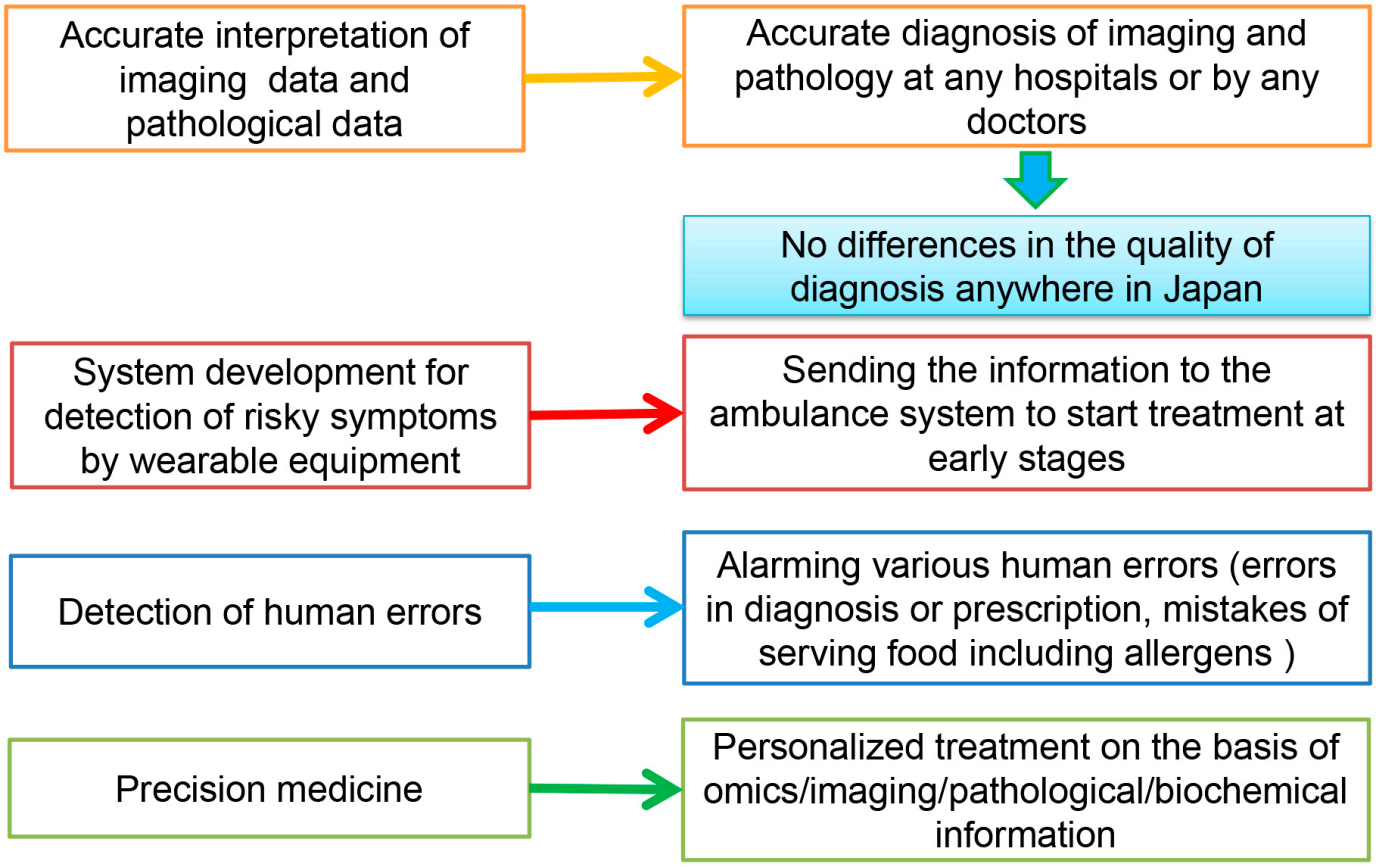
Summary of various subthemes in the “AI hospital” project and members participating in each subtheme project.

The content of the “AI Hospital” project consists of five subthemes, namely, A, B, C, D, and E, as shown in Figure 5 ^(1)^. Subtheme A is aiming to create a big medical database for developing AIs that can assist in diagnosis by finding relations between symptoms and diseases. This subproject includes construction of medical dictionaries (consisting of 400,000 terms) needed to translate speaking languages into text and aims to translate Japanese to English and other languages.

Subtheme B is committed to produce written documentation from a conversation/talk, such as conversations between doctors and patients in the examination room, and documentations of written nursing records and long-term care records are also taken into consideration. In addition, we are working on a system that automatically creates a summary at hospital admission or discharge from the electronic medical records, although it is not so easy for the Japanese language to do so. In particular, it is difficult to convert medical terms in speaking languages to “kanji,” the Japanese characters. It is also notable that the accuracy of conversion from spoken languages to text forms varies considerably because of the clarity of the words spoken by healthcare professionals. Our data indicated that when nurses obtained speaking training from a TV announcer, the conversion accuracy from spoken languages to text improved by 10% and reached more than 90%.

One group in subtheme C is developing AI-guided automated colonoscopy that finds the direction of the head of colonoscopy by recognizing space in colon and measures the pressure of the colorectal wall to avoid accidental penetration. The other is attempting the implementation of blood liquid biopsy for cancer screening, selection of molecular-targeted drug, monitoring minimum residual disease, and detection of relapse/recurrence by means of very highly sensitive detection of circulating tumor DNAs.

Although the members in subthemes A, B, and C are private companies, all members in subtheme D are hospitals. In subtheme D, five hospitals, namely, National Center for Child Health and Development, Keio University Hospital, Osaka University Hospital, Cancer Institute Hospital, and Yokosuka Kyosai Hospital, are evaluating various technologies mostly in collaboration with participants from other subthemes. In addition, to enhance international competitiveness of Japanese industries, including the participants of this project in medical fields, a team in subtheme E is designing various concepts/systems of “AI hospital” and is developing international strategies. Subtheme E is also trying to find solutions for regulatory issues or for standardization on how to implement the AI hospital systems. Because the information in the medical fields is extremely diverse, it is overly complicated to put them together in one system. For example, although hospitals have electronic medical record systems, their vendors are different, so it is quite difficult to unify them and make a single large database. However, we are creating a secure database that integrates various formats of medical records into one form and expect that AI based on multiple layers of information will provide various diagnostic measurements and will assist medical workers in the future.

## Medical AI Platform

**Figure 6.**
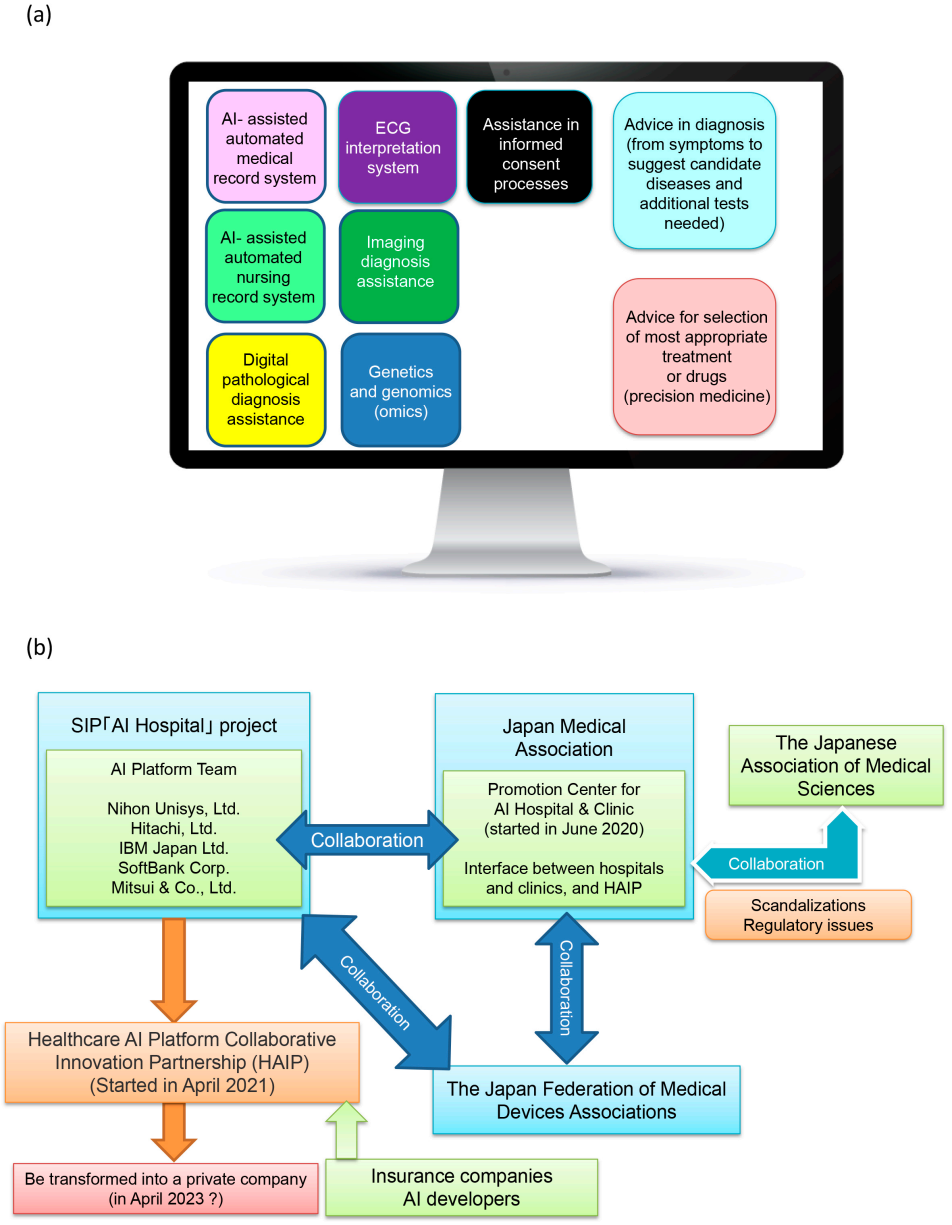
Construction of the “AI platform” and AI implementation network with Japan Medical Association. a. Images of the “AI platform.” The AI platform can provide various AI tools that are essential in medical fields. Because many AI tools can be put in this platform, the companies that develop AI tools can easily provide their services through this platform and doctors can easily find AI tools that they need through the AI platform. b. AI platform network. Five companies that participated in this project built “Health AI Platform Collaborative Innovation Partnership,” and they are collaborating to make an AI network with Promotion Center for AI Hospital & Clinic in Japan Medical Association. This network between the AI provider and AI users works together to address the issues that need the implementation of AIs in our health and medical care system.

One of the most important goals that we are aiming in this project is to build a platform that puts various AI tools in one place as shown in Figure 6. We are entering an era in which AI can detect abnormal lesions in ultrasound, CT, and endoscopic images as well as histopathological pictures with high accuracy. A 5G internet system will be widely implemented in the next few years, so the data-transfer speed will be improved to 100 times faster than the present speed, meaning that it will take less than a second to send high-resolution imaging data or pathological digital data from clinics/hospitals to the data server where various AI tools are provided and that doctors will have the accurate interpretation of them within a minute. If it happens, we believe that the diagnostic quality of various imaging, including CT and MRI, will be significantly improved anywhere in the world.

To make these AI tools widely available in clinical setting, we are collaborating with Japan Medical Association (JMA). To extract the problems or hurdles for AI implementation in clinics or hospitals, JMA established “Promotion Center for AI Hospital & Clinic” in June 2020, which is working with industries practically for the use of various AI tools through “Healthcare AI Platform Collaborative Innovation Partnership (HAIP)” that was established on April 1, 2021, as a spin-off organization of this project and was granted by the Minister of Health, Labour and Welfare as well as Minister of Economy, Trade and Industry. The HAIP is working not only on many practical issues for the implementation of various AI tools in the health and medical care system but also on standardization and regulatory issues for the use of AI tools in medical practices.

## Digitalization of Hospitals

In Japanese hospitals, the most common complaints are a long waiting time (waiting for examination, for laboratory tests, for imaging tests, for payment, etc.). I also have personal experiences that I had waited at the waiting room for nearly 1 h in spite that I went at the appointment time. Some hospitals provide you a buzzer when you come to the hospital and ring the buzzer when your doctor is ready, but if you do not know when the buzzer will sound (in 10 min or in 1 h), you even hesitate to go to the bathroom or tearoom. Many people now have smartphones, so if you can send a message to the patients (or family member) saying “We are sorry that your appointment is delayed by 10 minutes or 30 minutes,” the stress on patients will be significantly reduced. Patients fully understand that their appointment time may be delayed because of the longer medical examination of other patients

Patients also seem to wait for about 10-30 min for payment after their examination is over in many hospitals. If we can build a cashless system in hospitals, it will make patients less stressful. Furthermore, if doctors are able to send the prescription information directory to the pharmacy (although it is not allowed in Japan at present), patients can leave the hospital immediately after the doctor’s examination and pick up their medicine at the pharmacy that is close to their nearest station (autopayment and electric prescription). Considering the elevated risk of natural disasters such as earthquake or typhoon (and unusually heavy rainfall that often happens recently), it is extremely important for individual patients to keep their health and medical records in their hands, which assures continuous provision of medical care to individual patients under any conditions. If the health and medical records are reachable by individuals in any situations through individual smartphones, we are able to avoid treatment discontinuity. When this system is established, doctors can easily obtain the background status of patients, such as the history of their disease condition, even if they have any acute conditions during business trips or vacations.

## Conclusion

Many people worry that when AI is implemented into the clinical field, our humanity would be lost in the patient care system. However, I think that AI can provide sufficient time for a heartful human-to-human relationship, which is essential in the medical care. If you let AI and robots do what we must do for patients, but are doable without human involvement, we will be able to spend a huge amount of time for activities where human involvement is necessary. This will give you more time to look at and talk to patients and their family members with heartful interaction, restoring “empathy,” which is one of the fundamentals in medicine ^(9)^. I believe that AI-based and IT-based systems can bring “empathy” to the health medical care system.

## Article Information

### Conflicts of Interest

Y.N. is a stockholder and scientific advisor of OncoTherapy Science, but this work is not related to the company.
